# Supplementary material for: New targeted approaches for epigenetic age predictions
Source: BMC Biol. 2020 Jun 24;18:71. doi: 10.1186/s12915-020-00807-2 (PMC7315536; doi:10.1186/s12915-020-00807-2)
Supplement: Supplementary file 1 — Additional file 1: Figure S1. Demarcation of age-associated CpGs in Illumina BeadChip datasets. Figure S2. Comparison with epigenetic ageing clocks of Horvath and Hannum. Figure S3. Intersection of probes among different epigenetic ageing clocks. Figure S4. Selection of 6 CpG markers for pyrosequencing. Figure S5. Comparison of DNAm levels in pyrosequencing and BBA-seq. Figure S6. Comparison of age-associated DNAm in ddPCR versus BBA-seq. Figure S7. Correlation of DNAm with age in buccal swabs. Figure S8. Targeted sequences of pyrosequencing assays. Figure S9. Targeted sequences for BBA-seq. Table S1. DNAm profiles for candidate CpG selection. Table S2. Multivariable 65 CpG model for Illumina BeadChip data. Table S3. Primer list for Pyrosequencing assays. Table S4. Epigenetic aging signature based on pyrosequencing (6 CpG model). Table S5. Epigenetic aging signature based on pyrosequencing (9 CpG model). Table S6. Primer list for ddPCR assay. Table S7. Multivariable model for ddPCR (7 CpG). Table S8. Primer list for BBA-seq assay. Table S9. Multivariable model for BBA-seq of blood (9 CpG model). Table S10. Multivariable model for BBA-seq of buccal swabs (9 CpG model). Table S11. Machine learning model by Lasso (blood). Table S12. Machine learning model by ElasticNet (blood). Table S13. Machine learning model by Lasso (swab). Table S14. Machine learning model by ElasticNet (swab). [file 12915_2020_807_MOESM1_ESM.pdf]

Supplementary Materials

# New Targeted Approaches for Epigenetic Age Predictions

Yang Han, Julia Franzen, Thomas Stiehl, Michael Gobs, Chao-Chung Kuo, Miloš Nikolić, Jan Hapala, Barbara Elisabeth Koop, Klaus Strathmann, Stefanie Ritz-Timme, Wolfgang Wagner

**Table of contents**

Supplementary Figures ..... 2
Figure S1. Demarcation of age-associated CpGs in Illumina BeadChip datasets. ..... 2
Figure S2. Comparison with epigenetic ageing clocks of Horvath and Hannum. ..... 3
Figure S3. Intersection of probes among different epigenetic ageing clocks. ..... 4
Figure S4. Selection of 6 CpG markers for pyrosequencing. ..... 5
Figure S5. Comparison of DNAm levels in pyrosequencing and BBA-seq. ..... 6
Figure S6. Comparison of age-associated DNAm in ddPCR *versus* BBA-seq. ..... 6
Figure S7. Correlation of DNAm with age in buccal swabs. ..... 7
Figure S8. Targeted sequences of pyrosequencing assays. ..... 8
Figure S9. Targeted sequences for BBA-seq. ..... 10
Supplementary Tables ..... 11
Table S1. DNAm profiles for candidate CpG selection ..... 11
Table S2. Multivariable 65 CpG model for Illumina BeadChip data ..... 12
Table S3. Primer list for Pyrosequencing assays ..... 14
Table S4. Epigenetic aging signature based on pyrosequencing (6 CpG model) ..... 15
Table S5. Epigenetic aging signature based on pyrosequencing (9 CpG model) ..... 15
Table S6. Primer list for ddPCR assay ..... 16
Table S7. Multivariable model for ddPCR (7 CpG) ..... 17
Table S8. Primer list for BBA-seq assay ..... 18
Table S9. Multivariable model for BBA-seq of blood (9 CpG model) ..... 19
Table S10. Multivariable model for BBA-seq of buccal swabs (9 CpG model) ..... 19
Table S11. Machine learning model by Lasso (blood) ..... 20
Table S12. Machine learning model by ElasticNet (blood) ..... 20
Table S13. Machine learning model by Lasso (swab) ..... 21
Table S14. Machine learning model by ElasticNet (swab) ..... 21

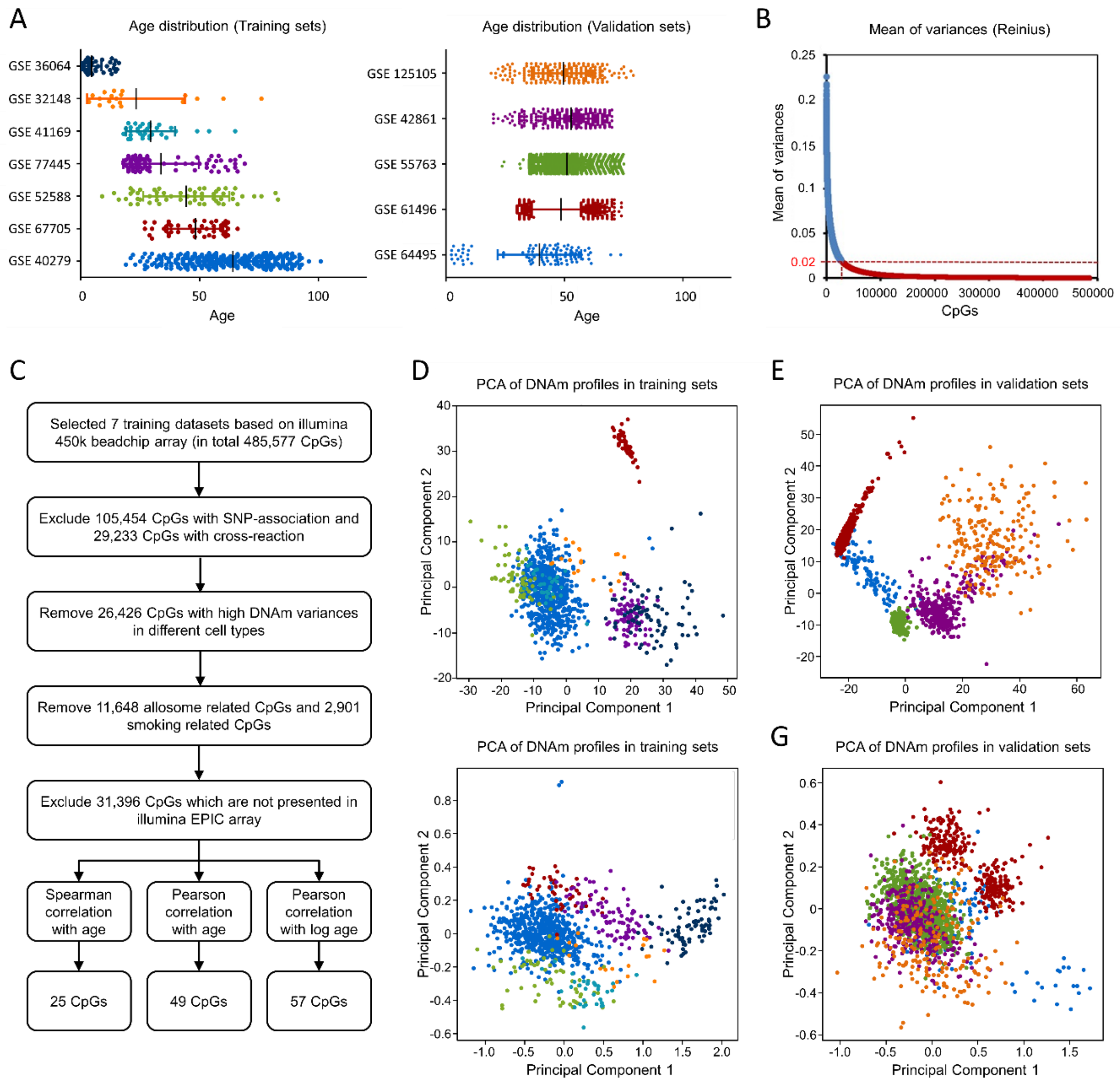


**Figure S1. Demarcation of age-associated CpGs in Illumina BeadChip datasets.**

**(A)** Age distribution of 450k Illumina BeadChip datasets in the 7 studies of the training and 5 studies of the validation cohorts (see also Supplemental Table S1; color code corresponds to Figures 1B,C, as well as to panels S1D-G, respectively). **(B)** To identify CpGs that are affected by the composition of leucocyte subsets we used the GEO datasets GSE35069 [42]. The variation of DNAm across the six leukocyte subsets was ranked and only CpGs below the threshold (R = 0.02; red dotted line) were considered for further analysis. **(C)** Schematic presentation how relevant age-associated CpGs were narrowed down. Smoking related , and SNP-associated and cross-reactive CpGs were excluded based on previous studies [39, 40, 43, 44]. **(D,E)** Principal component analysis (PCA) of all CpGs that are available for all samples of the training (385,587 CpGs) and validation (462,889 CpGs, dataset GSE55763 was reduced to 1000 samples) datasets demonstrated clear separation according to the different studies. This might be attributed to batch effects of Illumina BeadChip data. It is also conceivable that some differences are due to sampling, DNA isolation, bisulfite conversion, or hybridization in the different study groups. Therefore, correlation coefficients were determined separately for each study of the training set. **(F,G)** In the PCA of the 65 age-associated CpGs the separation of studies was less evident and particularly PC1 seemed to be influenced by donor age.

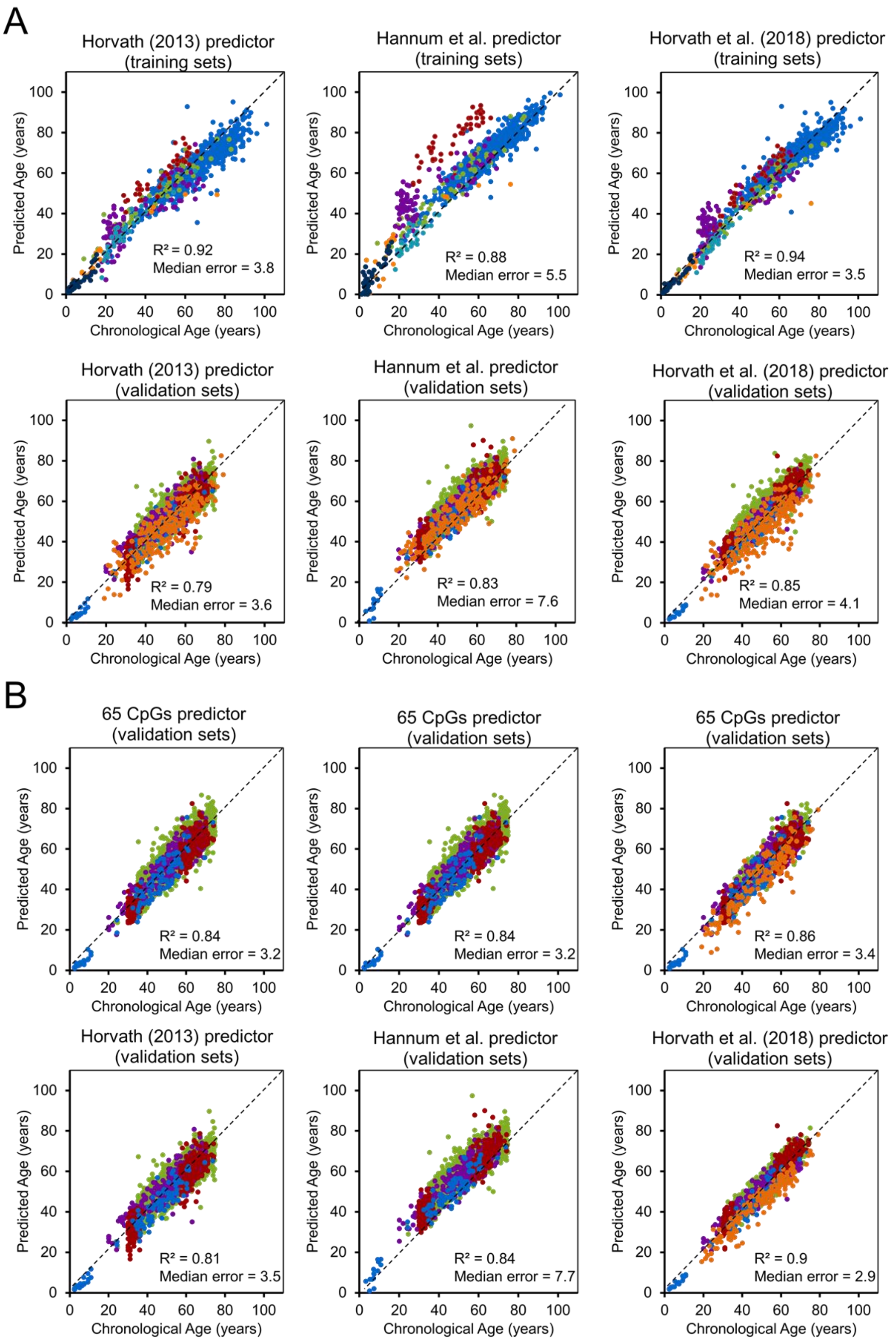


**Figure S2. Comparison with epigenetic ageing clocks of Horvath and Hannum.**

To better estimate the performance of our 65 CpG model (Figure 1B,C), we compared age-predictions using the previous described models by Horvath of 2013 [19], by Hannum et al. [17], and the Skin and Blood predictor of Horvath and colleagues of 2018 [46]. None of these alternative models outperformed our 65 CpG model in terms of median error (**A**). However, we noticed that in several datasets for one or few of the relevant CpGs of these alternative signatures DNAm values were not available. These missing values might

impact on the precision of age-predictions. We have therefore compared the age-predictions of our 65 CpG signature with the three alternative models only for those datasets of the validation set that contained beta-values for all relevant CpGs (GSE125105 was excluded and several samples with missing probes). The corresponding predictions with our 65 CpG model are always provided on top for each comparison **(B)**. Our 65 CpG model still provided better precision than the models of Horvarth 2013 and Hannum et al., 2013. However, the Skin and Blood predictor provided slightly better correlation and precision in this set of samples.

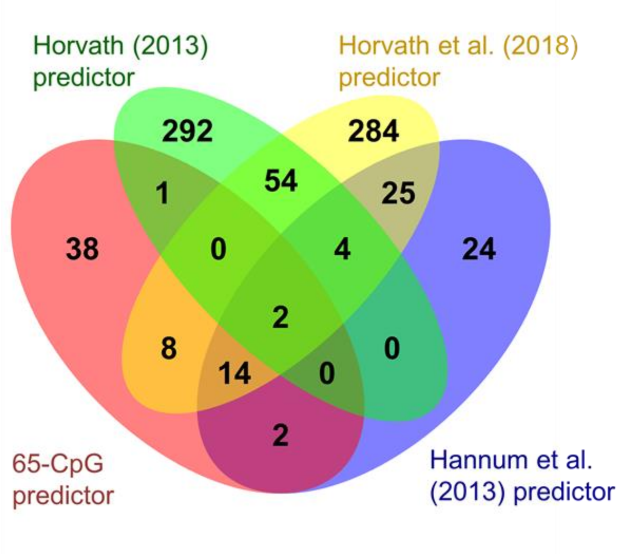


**Figure S3. Intersection of probes among different epigenetic ageing clocks.**

Venn diagram for the overlap of CpGs in our 65 CpG signature with the Horvath and Hannum clocks. The overlap with the Hannum clock [17] and Horvath et al. (2018) clock [46] was more pronounced.

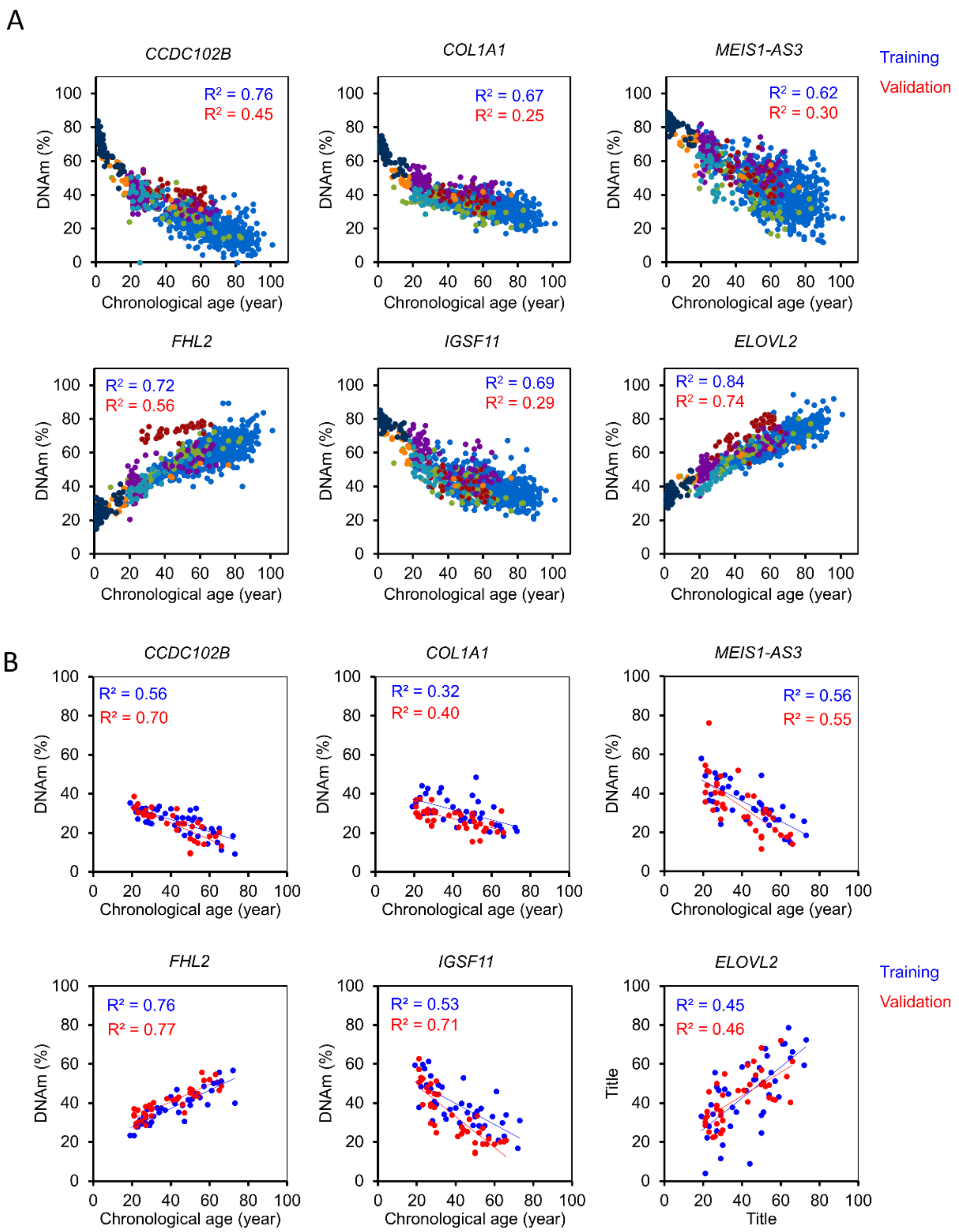


**Figure S4. Selection of 6 CpG markers for pyrosequencing.**

**(A)** DNAm levels of the Illumina BeadChip training sets were plotted for each of the 6 relevant CpGs in the genes *IGSF11*, *CCDC102B*, *FHL2*, *COL1A1*, *MEIS1-AS3* and *ELOVL2* against chronological age (color code as in Figure S1A). $R^2$ values are depicted for Illumina BeadChip training (blue) and validation sets (red). Please note that this correlation was significantly lower in the independent validation Illumina BeadChip datasets. **(B)** Pyrosequencing results for 40 blood samples of the training (blue) and 40 samples of the validation set (red).

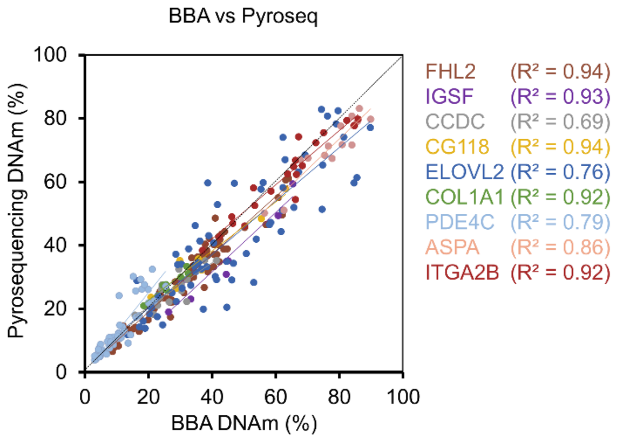


**Figure S5. Comparison of DNAm levels in pyrosequencing and BBA-seq.**

The DNA methylation levels at the nine age-associated genomic regions were compared in 15 blood samples with bisulfite barcoded amplicon sequencing (BBA-seq) and pyrosequencing. All CpGs that were covered by both types of measurements were included into the comparison.

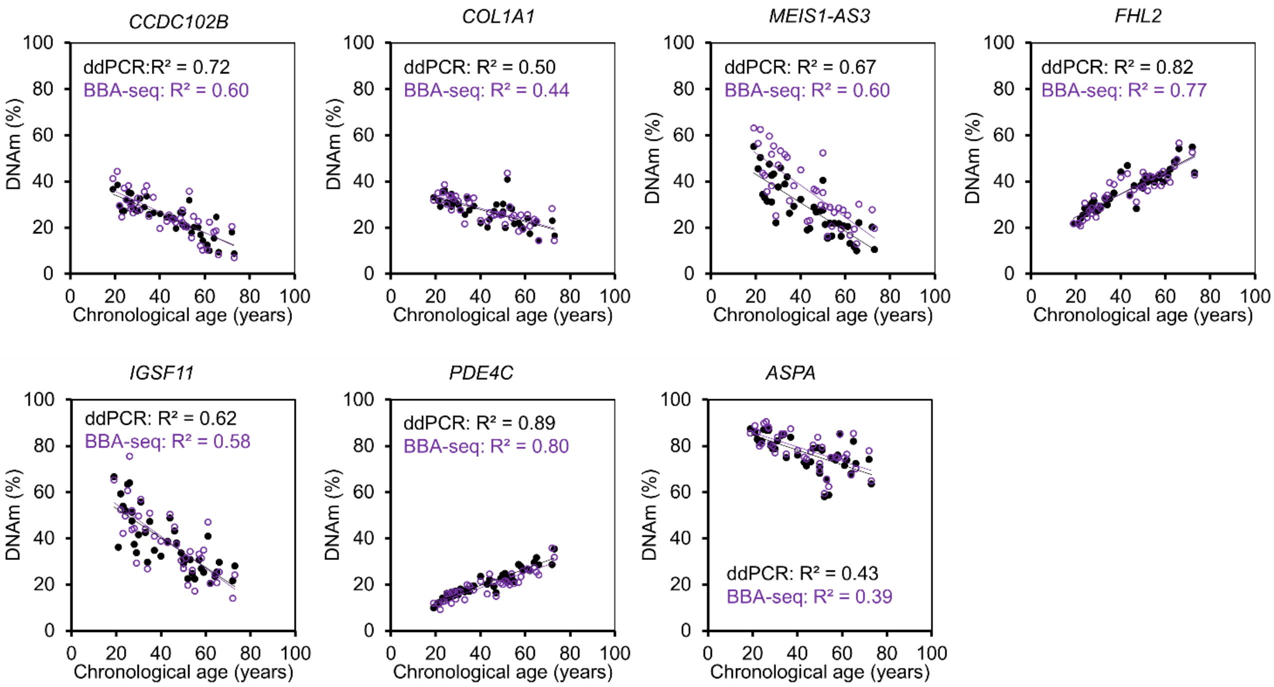


**Figure S6. Comparison of age-associated DNAm in ddPCR *versus* BBA-seq.**

DNAm measurements of the same CpGs by droplet digital PCR (ddPCR) (n = 40) and bisulfite barcoded amplicon sequencing (BBA-seq) (n = 38) were compared for blood samples of the training set at the same CpGs. The correlation with chronological age was consistently higher in ddPCR.

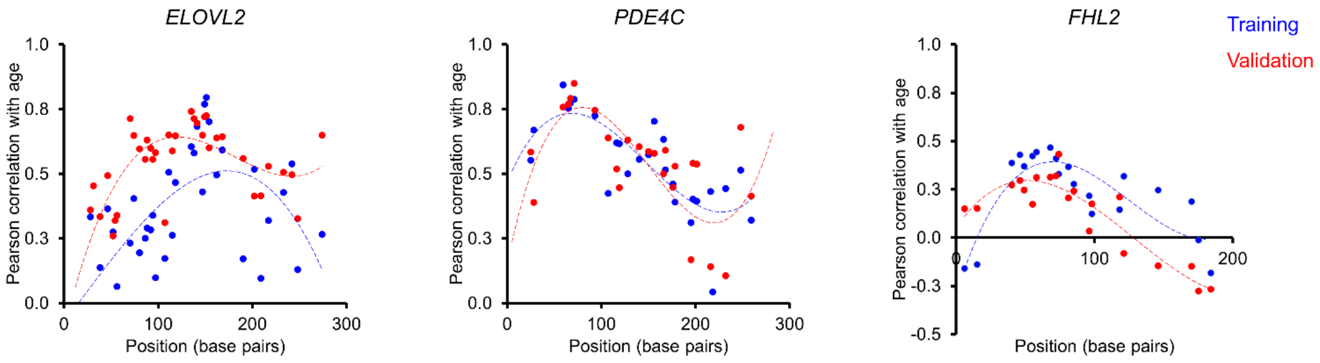


**Figure S7. Correlation of DNAm with age in buccal swabs.**

Pearson correlation of chronological age with the DNAm levels at the CpGs within amplicons of *ELOVL2*, *PDE4C* and *FHL2* in BBA-seq data of buccal swab samples.

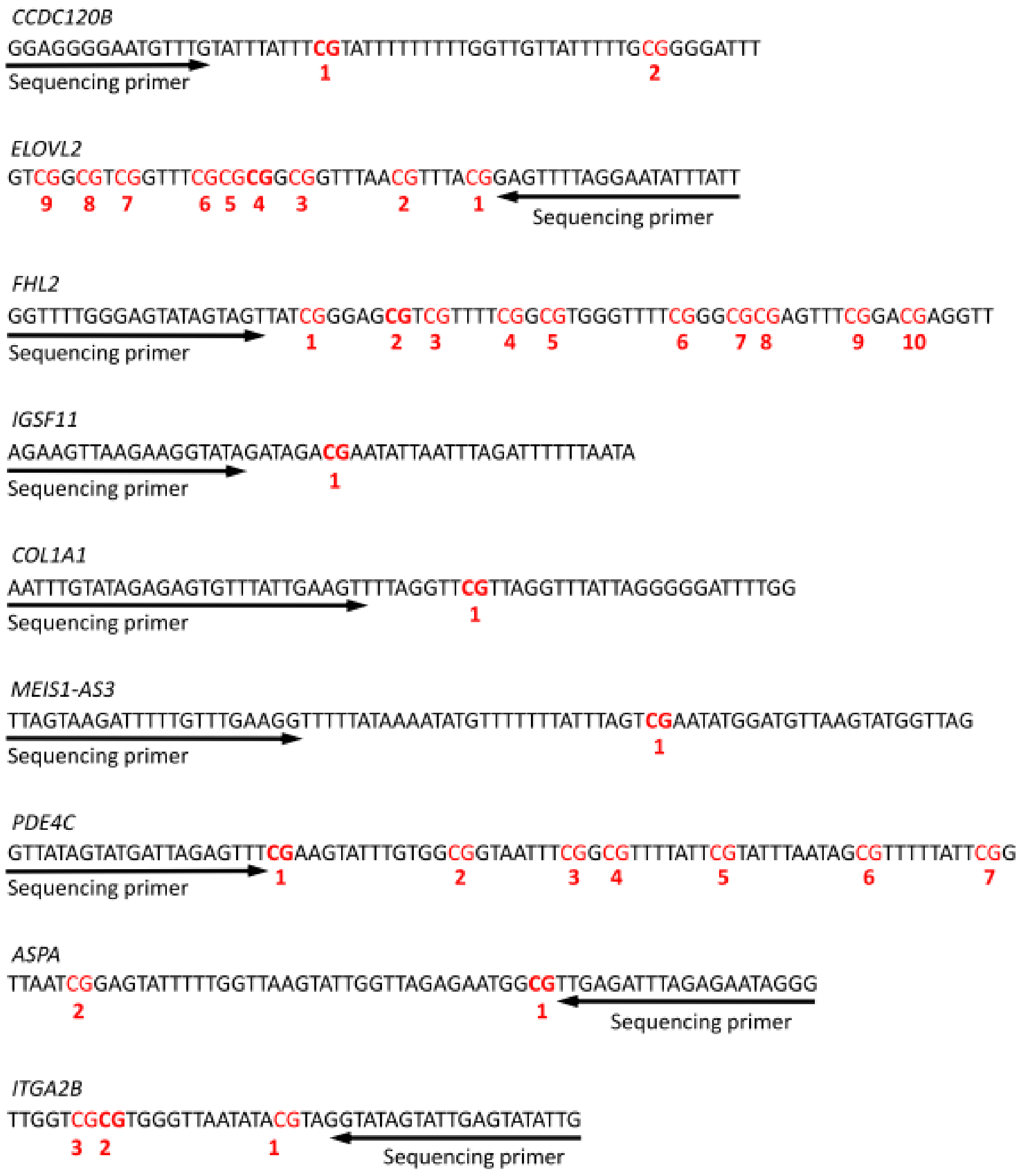

**Figure S8. Targeted sequences of pyrosequencing assays.**

Sequences for the nine genomic regions (Bisulfite treated sequences) for blood aging signature are depicted and CpG sites (red) are numbered by the dispensation order of pyrosequencing. The relevant CpGs with highest age-correlation are highlighted in bold.

**1. *CCDC102B* (chr18:66,389,223-66,389,599)**

AGTGGGGCAAGTACATGATACAAGGGAGGAAACAACCGAAGTAAGGGATTGTTCTGTGAAGTAAGAGAGTATTTTGTACAGGGTTACAAGC

CTTGCCCTGATGAAGCTTTATTCAAATGGCTTCTGAGAATTTTAGATCCGTAGTATTGTCTCTGGCTTTGAACGCTGTTGAGGGAGGGGAAT
140

GTTTGCACTCATCCCGCATCCTTTTTTGGCTGCTATCTTTGCGGGGATTGTTCAAGGAGAAATCCATCCTGACTGGAATGTAGTAAAGAAAG
198

GACAGTCATTCCCAGAGAAGGGCAATTTCCCCACTCTTCCCTTGTCGTTAAATGGTCAACTGAAGCTGCAGAAATGCTGATGATACAGACA

TTACTGGCCTG

**2. *MEIS1-AS3* (chr2:66,654,583-66,654,826)**

TTTGAATAATCAGTAAGATTTTTGCCTGAAGGTTTTCACAAAATATGCCCCTTTATCCAGTCGAACATGGATGCTAAGCATGGCCAGTGTGAC

TCATCCTGTTGCTTCAAAGGTAAATGTTTGCTATTAGTGACTAGAGCACGTTCGCCAGACCTCTTCCAGCACTCAGTACTTCAACTATTAGTT

TAGAATATATCAAACGTGTATGCACTTGTGATTAGTTCCTGGAAGAATTTTCTACATT
201

**3.*COL1A1* (chr17:48,275,263-48,275,502)**

TCTGTGTGTTTGTAGAAGGAGTATGAATCTGTATAGAGAGTGCTTACTGAAGCTCCAGGCTCGCCAGGCTCACCAGGGGGACCTTGGAAG
62

CCTTGGGGACCCTTGAGAAGAAGGAAAAAGATGGGTTAGAAGACAAGTCCCTGTCAACCTTCTCCAATCTTACCAAGAGATCTCTGAGCAT

CTCTCCTGCCCTCATCCCAGTCTTCCCTCCAAAAGACCAAAGCCCGAGGAGGCATATGA

**4.Elovl2 (chr6:11,044,729-11,045,049)**

GGGGAGGGGCGCAGGGCAAGTGAGGCGGCGCCCCCCGCCCCTGCGGCCTCGCGCGCCCCCTCCTGGGCGACCGACCTCGCCCTCG
44 50 68 72

CGTCCGCGGCGTCCCCTGCCGGCCGGGCGGCGATTTGCAGGTCCAGCCGGCGCCGGTTTCGCGCGGCGGCTCAACGTCCACGGAGC
90 92 95 109 113 133 136 145147149 152

CCCAGGAATACCCACCCGCTGCCCAGATCGGCAGCCGCTGCTGCGGGGAGAAGCAGTATCGTGCAGGGCGGGCACGCTGGTCTTGCTT

ACAGTTGGGCTTCGGTGGGTTTGAAGCACACATTAGGGGGAAATGGCTCTGTTCCTGCAGGT

**5. *FHL2* (chr2:106,015,702-106,015,913)**

CTTCGTGCCCTCCGGGTCTTGGGAGCACAGTAGTTATCGGGAGCGTCGCCTCCGGCGTGGGCTCTCGGGCGCGAGTTTCGGACGAGGC
38 56

CTGGGCGCGGTGGCAGGGGTCTGCCCACGCCGGGATCTCTGCCTGGTCCCAGGAGCGGGAGACTGGAGAAGCCCCAGGACGTGCCG

GGGGAGGCGGAGGGAGGAGGGGGTCACTTCTCAGGAGG

**6.*IGSF11* (chr3:118,706,493-118,706,721)**

GCCTTGAAAAAGACAAGGAAGAAAAAATGTAGCAGCAATTCACTAGGCATGTGACTTGAGATTTGGCAGGCTGCCTTGGCAGGGCTGGAC

AGTCTGTGGGCAGAAAATTCACATATTTTTTTCCTGCAGCCAGAAGCCAAGAAGGCACAGACAGACGAATACCAACTCAGATCCTTCAACA
156

AGCTAATAATCAATAAGACCTTCCTAAGGAGAACAATGAATGAATAAC

Figure is continued on next page

**7.*ASPA* (chr17:3,379,441-3,379,591)**

ACTACTTGGTGAAATGACTTCTTGTCACATTGCTGAAGAACATATACAAAAGGTTGCTATCTTTGGAGGAACCCATGGGAATGAGCTAAC**CG**
91
GAGTATTTCTGGTTAAGCATTGGCTAGAGAATGG**CG**CTGAGATTCAGAGAACAGGGCTG
127

**8.*ITGA2B* (chr17:42,467,630-42,467,778)**

CAATCCTTTTTGGGTGATGGAGCTCTTTAACCATTAAGACTTGATTCTGGTTGGGGGCTTTGCCTAGGGGAGCCTTCCCTGACTCCTCAGG

CTGGC**CGCG**TGGGCTAACACACGTAGGCACAGCATTGAGCACACTGTTTACTCTTGGT
97 99

**9.*PDE4C* (chr19:18,343,730-18,344,002)** (reverse sequence)

CATGGAGAACCTGGGGGTCGGCGAAGGGGCAGAGGCTTGCAGCAGGTTGAGT**CG**CTCT**CGCG**GC**CG**CCACAGCATGACCAGAGCCC**C**
53 59 61 65 87
**G**AAGCACCTGTGG**CG**GCAACCCCGG**CG**CCCCATC**CG**CATCCAACAGCGCTTCTATT**CG**GATCCGGACAAGTCCGCGGGCTGCCG**CG**AG
101 113 122 144 172
AGGGACCTGAGCCCGCGGCCGGAGCTCAGGAAGTCGCGGCTCTCCTGGCCCGTTTCCTCCTGCAGGCGGTAGGTGGC**CG**GGGCAGG
253

GGCCTCCTGCAG

**Figure S9. Targeted sequences for BBA-seq.**

Sequences for nine genomic regions that were analyzed by BBA-seq are depicted and all CpG sites are highlighted in red. The age-related CpGs selected by machine learning approach are highlighted in bold and by their ordering number in the sequences (underlined CpG was used in the multivariable linear model).

**Table S1. DNAm profiles for candidate CpG selection**

| GEO accession ID | Set used as | Tissue | Reference | Samples | Age (years) | Gender (f/m) |
|---|---|---|---|---|---|---|
| GSE 40279 | Training | Peripheral blood | [17] | 656 | 19-101 | 338/318 |
| GSE 67705 | Training | Peripheral blood | [63] | 44* | 27-66 | 2/44 |
| GSE 52588 | Training | Peripheral blood | [64] | 58 | 9-83 | 51/7 |
| GSE 77445 | Training | Peripheral blood | [65] | 85 | 18-69 | 42/43 |
| GSE 41169 | Training | Peripheral blood | [66] | 33 | 18-65 | 12/21 |
| GSE 32148 | Training | Peripheral blood | [67] | 19$ | 3.5-76 | 12/8 |
| GSE 36064 | Training | Peripheral blood | [45] | 78 | 1-15.3 | 0/78 |
| GSE 64495 | Validation | Peripheral blood | [68] | 106 | 2.3-73.7 | 76/37 |
| GSE 61496 | Validation | Peripheral blood | [69] | 310# | 30-74 | 148/164 |
| GSE 55763 | Validation | Peripheral blood | [70] | 2711 | 23.7-75 | 859/1805 |
| GSE 42861 | Validation | Peripheral blood | [71] | 335 | 20-70 | 239/96 |
| GSE125105 | Validation | Peripheral blood | - | 210 | 19-79 | 126/84 |

All datasets were generated on the 450k Illumina BeadChip. Data are accessible under http://www.ncbi.nlm.nih.gov/geo/.
* two, $ one and # two DNAm profiles of healthy donors were removed due to missing values for some CpGs.

**Table S2. Multivariable 65 CpG model for Illumina BeadChip data**

| Target site | Gene Name | CHR | Map Info | Coeffients | P value |
|---|---|---|---|---|---|
| (Intercept) | | | | 0.71 | 0.0284 |
| cg19283806 | *CCDC102B* | 18 | 66389420 | -0.59 | 0.0019 |
| cg11807280 | *MEIS1-AS3* | 2 | 66654644 | -0.21 | 0.0488 |
| cg00329615 | *IGSF11* | 3 | 118706648 | 0.01 | 0.9405 |
| cg22454769 | *FHL2* | 2 | 106015767 | 0.05 | 0.8418 |
| cg16867657 | *ELOVL2* | 6 | 11044877 | 2.15 | 0.0000 |
| cg22796704 | *ARHGAP22* | 10 | 49673534 | -0.69 | 0.0089 |
| cg09809672 | *EDARADD* | 1 | 236557682 | -0.64 | 0.0018 |
| cg18618815 | *COL1A1* | 17 | 48275324 | -0.77 | 0.0107 |
| cg25533247 | *AKAP8L* | 19 | 15530630 | 0.12 | 0.7874 |
| cg02286081 | *HLA-DPB1* | 6 | 33043841 | -0.23 | 0.3118 |
| cg20222376 | *AKAP8L* | 19 | 15530606 | 0.003 | 0.9949 |
| cg19344626 | *NWD1* | 19 | 16830749 | -0.06 | 0.8783 |
| cg07082267 | | 16 | 85429035 | -0.22 | 0.6192 |
| cg15845821 | *NWD1* | 19 | 16830613 | 1.54 | 0.0001 |
| cg11741201 | *FJX1* | 11 | 35638398 | 0.34 | 0.2894 |
| cg16054275 | *F5* | 1 | 169556022 | 0.19 | 0.6404 |
| cg18933331 | | 1 | 110186418 | -0.41 | 0.2116 |
| cg20249566 | *NWD1* | 19 | 16830739 | -0.78 | 0.0897 |
| cg16604658 | *TBK1* | 12 | 64847188 | 0.50 | 0.0576 |
| cg07583137 | *CHMP4C* | 8 | 82644012 | 0.06 | 0.7619 |
| cg16008966 | | 1 | 114761794 | -0.39 | 0.1889 |
| cg14556683 | *EPHX3* | 19 | 15342982 | 0.10 | 0.7028 |
| cg03746976 | *C16orf57* | 16 | 58035805 | 0.02 | 0.9569 |
| cg14314729 | | 5 | 179815975 | 0.27 | 0.4216 |
| cg03431918 | | 17 | 77716367 | -0.26 | 0.5443 |
| cg22156456 | *EIF1* | 17 | 39844239 | 0.25 | 0.5051 |
| cg23078123 | *GPR177* | 1 | 68577796 | -0.73 | 0.0174 |
| cg09748749 | *ASL* | 7 | 65540429 | -1.14 | 0.0001 |
| cg17457912 | *C17orf91* | 17 | 1617102 | -0.06 | 0.8868 |
| cg06492796 | | 12 | 96883057 | -0.20 | 0.4626 |
| cg17593342 | | 6 | 14037614 | 0.86 | 0.0011 |
| cg05308819 | | 1 | 155959156 | -0.89 | 0.0632 |
| cg22512670 | *RPS6KA1* | 1 | 26855765 | -0.33 | 0.3007 |
| cg01820962 | *NT5DC1* | 6 | 116511817 | -0.85 | 0.0000 |
| cg06639320 | *FHL2* | 2 | 106015739 | 1.92 | 0.0000 |
| cg03224418 | *SAMD10;PRPF6* | 20 | 62611858 | 0.92 | 0.0030 |
| cg17436656 | *RARG* | 12 | 53627106 | -0.07 | 0.7846 |
| cg19500607 | *HTR4* | 5 | 148034319 | 0.52 | 0.0392 |
| cg03735592 | *NHSL1* | 6 | 138821354 | 0.32 | 0.3261 |
| cg20669012 | | 3 | 11102341 | 0.38 | 0.3835 |

| | | | | | |
|---|---|---|---|---|---|
| cg19761273 | *CSNK1D* | 17 | 80232096 | 0.03 | 0.9316 |
| cg07080372 | *SLC25A22* | 11 | 796607 | -1.46 | 0.0001 |
| cg03638795 | *SIGIRR* | 11 | 416499 | -0.27 | 0.3464 |
| cg19722847 | *IPO8* | 12 | 30849114 | -0.59 | 0.1894 |
| cg24711336 | | 10 | 80063791 | 0.64 | 0.0533 |
| cg26935102 | *POLR3GL;ANKRD34A* | 1 | 145470946 | 0.47 | 0.2069 |
| cg10221746 | | 1 | 156629412 | 0.27 | 0.4315 |
| cg02085953 | *ARID5A* | 2 | 97202260 | -0.64 | 0.1876 |
| cg04604946 | *LRRC23* | 12 | 7023352 | -1.11 | 0.0066 |
| cg08558886 | | 2 | 151469837 | -0.17 | 0.4577 |
| cg22361181 | *NKIRAS2* | 17 | 40171740 | 0.66 | 0.2161 |
| cg04208403 | *ZNF423* | 16 | 49525807 | -0.09 | 0.8077 |
| cg12623930 | *ABHD14B* | 3 | 52008802 | 0.09 | 0.8599 |
| cg21572722 | *ELOVL2* | 6 | 11044894 | -0.43 | 0.3779 |
| cg17885226 | | 6 | 105388731 | 0.33 | 0.3176 |
| cg00748589 | | 12 | 11653486 | 2.54 | 0.0000 |
| cg13033938 | *IP6K1* | 3 | 49824475 | -3.63 | 0.0000 |
| cg19784428 | *NWD1* | 19 | 16830746 | 0.10 | 0.8084 |
| cg22016779 | *DNER* | 2 | 230452311 | -0.43 | 0.1755 |
| cg01974375 | *PI4KB* | 1 | 151298954 | -0.41 | 0.3780 |
| cg25256723 | *F5* | 1 | 169555944 | -0.27 | 0.4739 |
| cg24724428 | *ELOVL2* | 6 | 11044888 | 0.70 | 0.0009 |
| cg07547549 | *SLC12A5* | 20 | 44658225 | -0.11 | 0.7152 |
| cg25410668 | *RPA2* | 1 | 28241577 | 0.28 | 0.2035 |
| cg21296230 | *GREM1* | 15 | 33010536 | 0.93 | 0.0015 |

This model was trained for transformed age instead of chronological age, as described before [19].

**Table S3. Primer list for Pyrosequencing assays**

| Primer | Sequence |
|---|---|
| ***CCDC102B*** | |
| Forward | 5'- TGTTGAGGGAGGGGAATGTTTGTATTTAT-3' |
| Reverse | 5'-Biotin- CCAATAATATCTATATCATCAACATTTCTACAACTT-3' |
| Sequencing | 5'- GGAGGGGAATGTTTG -3' |
| ***IGSF11*** | |
| Forward | 5'- GTTGGATAGTTTGTGGGTAGAAAATTTA -3' |
| Reverse | 5'-Biotin- ATTATTCATTCATTATTCTCCTTAAAAAAATCTTATT -3' |
| Sequencing | 5'- AGAAGTTAAGAAGGTATAGATA -3' |
| ***ELOVL2*** | |
| Forward | 5'-Biotin- GGGAGGGGAGTAGGGTAAGTGA -3' |
| Reverse | 5'- CCATCTAAACAACCAATAAATATTCCTAAAAC -3' |
| Sequencing | 5'- AATAAATATTCCTAAAACTC -3' |
| ***COL1A1*** | |
| Forward | 5'- TTGAAGGGAAGAGGTAAGGAAGATTTTA -3' |
| Reverse | 5'- Biotin- TAACCCATCTTTTTCCTTCTTCTCA -3' |
| Sequencing | 5'- AATTTGTATAGAGAGTGTTTATTG -3' |
| ***MEIS1-AS3*** | |
| Forward | 5'- TTGAATAATTAGTAAGATTTTTGTTTGAAGGTTT -3' |
| Reverse | 5'-Biotin- TTACCTTTAAAACAACAAAATAAATCACACTAACC -3' |
| Sequencing | 5'- TTAGTAAGATTTTTGTTTG -3' |
| ***FHL2*** | |
| Forward | 5'- GTGTTTTTAGGGTTTTGGGAGTATAGTAGT -3' |
| Reverse | 5'-Biotin- CACCTCCTAAAACTTCTCCAATCTCC -3' |
| Sequencing | 5'-TATTTTTTAAGGTAGTAAGAGT-3' |
| ***ASPA*** | |
| Forward | 5'-Biotin- ATTATTTGGTGAAATGATT -3' |
| Reverse | 5'- CAACCCTATTCTCTAAATCTC -3' |
| Sequencing | 5'- CCCTATTCTCTAAATCTCA -3' |
| ***ITGA2B*** | |
| Forward | 5'-Biotin- TAATTTTTTTTGGGTGATG -3' |
| Reverse | 5'- ACCAAAAATAAACAATATACTCAAT -3' |
| Sequencing | 5'- CAATATACTCAATACTATACCT -3' |
| ***PDE4C*** | |
| Forward | 5'- AGGTTTGTAGTAGGTTGAG -3' |
| Reverse | 5'-Biotin- AACTCAAATCCCTCTC -3' |
| Sequencing | 5'- GTTATAGTATGATTAGAGTTT -3' |

**Table S4. Epigenetic aging signature based on pyrosequencing (6 CpG model)**

| Target site | Gene Name | CHR | Map Info | Coeffients | P value |
|---|---|---|---|---|---|
| (Intercept) | | | | 32.07 | 0.0161 |
| cg22454769 | *FHL2* | 2 | 106015767 | 0.99 | 0.0001 |
| cg00329615 | *IGSF11* | 3 | 118706648 | -0.12 | 0.3512 |
| cg19283806 | *CCDC102B* | 18 | 66389420 | -0.99 | 0.0002 |
| cg11807280 | *MEIS1-AS3* | 2 | 66654644 | -0.09 | 0.5081 |
| cg16867657 | *ELOVL2* | 6 | 11044877 | 0.05 | 0.4630 |
| cg18618815 | *COL1A1* | 17 | 48275324 | 0.11 | 0.5411 |

**Table S5. Epigenetic aging signature based on pyrosequencing (9 CpG model)**

| Target site | Gene Name | CHR | Map Info | Coeffients | P value |
|---|---|---|---|---|---|
| (Intercept) | | | | 37.83 | 0.0107 |
| cg22454769 | *FHL2* | 2 | 106015767 | 0.53 | 0.0236 |
| cg00329615 | *IGSF11* | 3 | 118706648 | -0.17 | 0.1289 |
| cg19283806 | *CCDC102B* | 18 | 66389420 | -0.29 | 0.2604 |
| cg11807280 | *MEIS1-AS3* | 2 | 66654644 | 0.02 | 0.8275 |
| cg16867657 | *ELOVL2* | 6 | 11044877 | 0.12 | 0.0718 |
| cg18618815 | *COL1A1* | 17 | 48275324 | -0.1 | 0.6015 |
| cg02228185 | *ASPA* | 17 | 3379567 | -0.19 | 0.1153 |
| cg25809905 | *ITGAB* | 17 | 42467728 | -0.22 | 0.0610 |
| NA[1] | *PDE4C* | 19 | 18343915 | 0.93 | 0.0030 |

[1] A neighboring CpG was used, which is not included on the Illumina BeadChip.

**Table S6. Primer list for ddPCR assay**

| Primer | Sequence |
|---|---|
| ***CCDC102B*** | |
| Forward | 5'- AGTATTGTTTTTGGTTTTGAA-3' |
| Reverse | 5'- CCCTTCTCTAAAAATAACTATCC-3' |
| Probe | 6-Fam - AGGGAGGGGAATGTTTGTATTTATTTCGTA -BHQ-1[a] |
| | Hex-AGGGAGGGGAATGTTTGTATTTATTTTGTA -BHQ-1[b] |
| ***COL1A1*** | |
| Forward | 5'-AGGAGAGTTTGTGTGTTTGT-3' |
| Reverse | 5'-TCTAACCCATCTTTTTCCTTCT-3' |
| Probe | 6-Fam -TGAAGTTTTAGGTTCGTTAGGTTTATTAGG - BHQ-1[a] |
| | Hex -TGAAGTTTTAGGTTTGTTAGGTTTATTAGG - BHQ-1[b] |
| ***MEIS1-AS3*** | |
| Forward | 5'-AGAGTAYGTTYGTTAGATTT-3' |
| Reverse | 5'- AAATCCTCATAACAATAACTTAAAA-3' |
| Probe | 6-Fam - AGAATATATTAAACGTGTATGTATTTGTGA - BHQ-1[a] |
| | Hex - AGAATATATTAAATGTGTATGTATTTGTGA - BHQ-1[b] |
| ***FHL 2*** | |
| Forward | 5'-TATTTTTTGTTTGTTAGGGTTTTT-3' |
| Reverse | 5'-TCCTAAAACCAAACAAAAATCC-3' |
| Probe | 6-Fam -TTTTGGGAGTATAGTAGTTATCGGGAG - BHQ-1[a] |
| | Hex -TTTTGGGAGTATAGTAGTTATTGGGAG - BHQ-1[b] |
| ***ASPA*** | |
| Forward | 5'-AGGTTGTTATTTTTGGAGGA-3' |
| Reverse | 5'-CCTCCAACCCTATTCTCTAA-3' |
| Probe | 6-Fam -TGGGAATGAGTTAATCGGAGTAT- BHQ-1[a] |
| | Hex -TGGGAATGAGTTAATTGGAGTAT- BHQ-1[b] |
| ***IGSF11*** | |
| Forward | 5'-TGGTAGGGTTGGATAGTT-3' |
| Reverse | 5'-AATTATTCATTCATTATTCTCCTTAA-3' |
| Probe | 6-Fam -AAGGTATAGATAGACGAATATTAATTTAGA- BHQ-1[a] |
| | Hex -AAGGTATAGATAGATGAATATTAATTTAGA- BHQ-1[b] |
| ***PDE4C*** | |
| Forward | 5'-GAGGTTTGTAGTAGGTTGAGT-3' |
| Reverse | 5'-CRAACTCAAATCCCTCTCR-3' |
| Probe | 6-Fam -TAGTATGATTAGAGTTTCGAAGTATTTGTG- BHQ-1[a] |
| | Hex -TAGTATGATTAGAGTTTTGAAGTATTTGTG- BHQ-1[b] |

[a] Probe targeting the methylated sequence.
[b] Probe targeting the non-methylated sequence.

**Table S7. Multivariable model for ddPCR (7 CpG)**

| Target site | Gene Name | CHR | Map Info | Coeffients | P value |
|---|---|---|---|---|---|
| (Intercept) | | | | 28.52 | 0.1253 |
| cg06639320 | *FHL2* | 2 | 106015739 | 0.4 | 0.0396 |
| cg00329615 | *IGSF11* | 3 | 118706648 | -0.14 | 0.0995 |
| cg19283806 | *CCDC102B* | 18 | 66389420 | -0.41 | 0.0246 |
| NA[1] | *MEIS1-AS3* | 2 | 66654782 | -0.02 | 0.8712 |
| cg18618815 | *COL1A1* | 17 | 48275324 | 0.01 | 0.9676 |
| NA[1] | *ASPA* | 17 | 3379531 | -0.12 | 0.3723 |
| NA[1] | *PDE4C* | 19 | 18343915 | 1.22 | 0.0002 |

[1] A neighboring CpG was used, which is not included on the Illumina BeadChip.

**Table S8. Primer list for BBA-seq assay**

| Primer | Sequence |
|---|---|
| ***CCDC102B*** | |
| Forward | 5'-CTCTTTCCCTACACGACGCTCTTCCGATCTAGTGGGGTAAGTATATGATATAAGGGAGGAAATA -3' |
| Reverse | 5'- CTGGAGTTCAGACGTGTGCTCTTCCGATCTCAAACCAATAATATCTATATCATCAACATTTCT -3' |
| ***IGSF11*** | |
| Forward | 5'- CTCTTTCCCTACACGACGCTCTTCCGATCTGTTTTGAAAAAGATAAGGAAGAAAAAATGTAGTA-3' |
| Reverse | 5'- CTGGAGTTCAGACGTGTGCTCTTCCGATCTATTATTCATTCATTATTCTCCTTAAAAAAATCTTATT -3' |
| ***ELOVL2*** | |
| Forward | 5'- CTCTTTCCCTACACGACGCTCTTCCGATCTGGGGAGGGGAGTAGGGTAAGTGA-3' |
| Reverse | 5'- CTGGAGTTCAGACGTGTGCTCTTCCGATCTACCTACAAAAACAAAACCATTTCCCCCTAATAT-3' |
| ***COL1A1*** | |
| Forward | 5'- CTCTTTCCCTACACGACGCTCTTCCGATCTTTTGTGTGTTTGTAGAAGGAGTATGAATTTGTATAG -3' |
| Reverse | 5'- CTGGAGTTCAGACGTGTGCTCTTCCGATCTTCATATACCTCCTCCAACTTTAATCTTTTAAA-3' |
| ***MEIS1-AS3*** | |
| Forward | 5'- CTCTTTCCCTACACGACGCTCTTCCGATCTTTTGAATAATTAGTAAGATTTTTGTTTGAAGGTTT-3' |
| Reverse | 5'- CTGGAGTTCAGACGTGTGCTCTTCCGATCTAATATAAAAAATTCTTCCAAAAACTAATCACAAATACA -3' |
| ***FHL2*** | |
| Forward | 5'- CTCTTTCCCTACACGACGCTCTTCCGATCTTTTAGTGTTTTTAGGGTTTTGGGAGTATAGTAGTT-3' |
| Reverse | 5'- CTGGAGTTCAGACGTGTGCTCTTCCGATCTCCTCCTAAAAAATAACCCCCTCCTCCCT-3' |
| ***ASPA*** | |
| Forward | 5'-CTCTTTCCCTACACGACGCTCTTCCGATCTATTATTTGGTGAAATGATT-3' |
| Reverse | 5'- CTGGAGTTCAGACGTGTGCTCTTCCGATCTCAACCCTATTCTCTAAATCTC -3' |
| ***ITGA2B*** | |
| Forward | 5'-CTCTTTCCCTACACGACGCTCTTCCGATCTTAATTTTTTTTGGGTGATG -3' |
| Reverse | 5'- CTGGAGTTCAGACGTGTGCTCTTCCGATCTACCAAAAATAAACAATATACTCAAT-3' |
| ***PDE4C*** | |
| Forward | 5'- CTCTTTCCCTACACGACGCTCTTCCGATCTTATGGAGAATTTGGGG -3' |
| Reverse | 5'- CTGGAGTTCAGACGTGTGCTCTTCCGATCTCTACAAAAAACCCCTACC -3' |
| ***CD6*** | |
| Forward | 5'- CTCTTTCCCTACACGACGCTCTTCCGATCTAGTATAGGTAGTTGGGGTTTTTTTATTAGTTTTTGTA-3' |
| Reverse | 5'- CTGGAGTTCAGACGTGTGCTCTTCCGATCTCCAAATCTACTCTACCCTTTACTATTCTTATTCCTAT-3' |
| ***SERPINB5*** | |
| Forward | 5'- CTCTTTCCCTACACGACGCTCTTCCGATCTATTGTGGATAAGTTGTTAAGAGGTTTGAGTAGG-3' |
| Reverse | 5'- CTGGAGTTCAGACGTGTGCTCTTCCGATCTAAACAAACAAACCAAAAACACAAAAACCTAAATAT-3' |

**Table S9. Multivariable model for BBA-seq of blood (9 CpG model)**

| Target site | Gene Name | CHR | Map Info | Coeffients | P value |
|---|---|---|---|---|---|
| (Intercept) | | | | 42.61 | 0.0082 |
| NA[1] | *FHL2* | 2 | 106015747 | 0.19 | 0.4315 |
| cg00329615 | *IGSF11* | 3 | 118706648 | -0.12 | 0.1238 |
| cg19283806 | *CCDC102B* | 18 | 66389420 | -0.15 | 0.2637 |
| NA[1] | *MEIS1-AS3* | 2 | 66654782 | -0.07 | 0.5082 |
| cg16867657 | *ELOVL2* | 6 | 11044877 | 0.41 | 0.0153 |
| cg18618815 | *COL1A1* | 17 | 48275324 | -0.17 | 0.2692 |
| NA[1] | *ASPA* | 17 | 3379531 | -0.11 | 0.3643 |
| NA[1] | *ITGAB* | 17 | 42467726 | -0.21 | 0.0662 |
| NA[1] | *PDE4C* | 19 | 18343886 | 0.82 | 0.0060 |

[1] A neighboring CpG was used, which is not included on the Illumina BeadChip.

**Table S10. Multivariable model for BBA-seq of buccal swabs (9 CpG model)**

| Target site | Gene Name | CHR | Map Info | Coeffients | P value |
|---|---|---|---|---|---|
| (Intercept) | | | | 24.10 | 0.4794 |
| NA[1] | *FHL2* | 2 | 106015747 | 0.68 | 0.0248 |
| cg00329615 | *IGSF11* | 3 | 118706648 | -0.07 | 0.6255 |
| cg19283806 | *CCDC102B* | 18 | 66389420 | 0.07 | 0.5596 |
| NA[1] | *MEIS1-AS3* | 2 | 66654782 | 0.26 | 0.2405 |
| cg16867657 | *ELOVL2* | 6 | 11044877 | 0.53 | 0.0004 |
| cg18618815 | *COL1A1* | 17 | 48275324 | -0.02 | 0.9299 |
| NA[1] | *ASPA* | 17 | 3379531 | -0.40 | 0.0155 |
| NA[1] | *ITGAB* | 17 | 42467726 | -0.42 | 0.2493 |
| NA[1] | *PDE4C* | 19 | 18343886 | 0.69 | 0.0001 |

[1] A neighboring CpG was used, which is not included on the Illumina BeadChip.

**Table S11. Machine learning model by Lasso (blood)**

| | Target sites* | Coefficients |
|---|---|---|
| | (Intercept) | 14.02 |
| 1 | *ASPA*.093 | -0.15 |
| 2 | *CCDC102B*.142 | -0.38 |
| 3 | *CCDC102B*.200 | -0.04 |
| 4 | *MEIS1-AS3*.203 | -0.09 |
| 5 | *COL1A1*.063 | -0.04 |
| 6 | *Elovl2*.074 | 0.10 |
| 7 | *Elovl2*.092 | 0.08 |
| 8 | *Elovl2*.115 | 0.28 |
| 9 | *Elovl2*.151 | 0.44 |
| 10 | *FHL2*.146 | 0.39 |
| 11 | *ITGA2B*.099 | -0.11 |
| 12 | *PDE4C*.059 | 0.16 |
| 13 | *PDE4C*.067 | 0.24 |
| 14 | *PDE4C*.071 | 0.18 |
| 15 | *PDE4C*.093 | 0.30 |
| 16 | *PDE4C*.119 | 0.33 |
| 17 | *PDE4C*.128 | 0.13 |

* Target sites correspond to the CpG# in Supplementary Figure S9.

**Table S12. Machine learning model by ElasticNet (blood)**

| | Target sites* | Coefficients |
|---|---|---|
| | (Intercept) | 17.46 |
| 1 | *ASPA*.093 | -0.16 |
| 2 | *CCDC102B*.142 | -0.35 |
| 3 | *CCDC102B*.200 | -0.01 |
| 4 | *MEIS1-AS3*.064 | -0.02 |
| 5 | *MEIS1-AS3*.203 | -0.07 |
| 6 | *COL1A1*.063 | -0.07 |
| 7 | *Elovl2*.070 | 0.39 |
| 8 | *Elovl2*.092 | 0.03 |
| 9 | *Elovl2*.115 | 0.26 |
| 10 | *Elovl2*.135 | 0.08 |
| 11 | *Elovl2*.138 | 0.03 |
| 12 | *Elovl2*.147 | 0.02 |
| 13 | *Elovl2*.149 | 0.04 |
| 14 | *Elovl2*.151 | 0.25 |
| 15 | *Elovl2*.154 | 0.06 |
| 16 | *FHL2*.040 | 0.02 |
| 17 | *FHL2*.058 | 0.02 |
| 18 | *FHL2*.146 | 0.34 |
| 19 | *ITGA2B*.099 | -0.15 |
| 20 | *PDE4C*.059 | 0.14 |
| 21 | *PDE4C*.065 | 0.10 |
| 22 | *PDE4C*.067 | 0.20 |
| 23 | *PDE4C*.071 | 0.17 |
| 24 | *PDE4C*.093 | 0.25 |
| 25 | *PDE4C*.119 | 0.32 |
| 26 | *PDE4C*.128 | 0.14 |

* Target sites correspond to the CpG# in Supplementary Figure S9.

**Table S13. Machine learning model by Lasso (swab)**

| | Target sites* | Coefficients |
|---|---|---|
| | (Intercept) | -15.27 |
| 1 | *ASPA*.129 | -0.16 |
| 2 | *CCDC102B*.323 | -0.06 |
| 3 | *MEIS1-AS3*.144 | 0.17 |
| 4 | *MEIS1-AS3*.203 | 0.08 |
| 5 | *Elovl2*.046 | 0.06 |
| 6 | *Elovl2*.052 | 0.64 |
| 7 | *Elovl2*.094 | -0.06 |
| 8 | *Elovl2*.097 | -0.07 |
| 9 | *Elovl2*.111 | 0.03 |
| 10 | *Elovl2*.135 | 0.32 |
| 11 | *Elovl2*.149 | 0.29 |
| 12 | *Elovl2*.151 | 0.12 |
| 13 | *Elovl2*.154 | 0.10 |
| 14 | *Elovl2*.202 | 0.02 |
| 15 | *Elovl2*.233 | 0.05 |
| 16 | *Elovl2*.248 | -0.01 |
| 17 | *FHL2*.058 | 0.11 |
| 18 | *FHL2*.072 | 0.42 |
| 19 | *ITGA2B*.099 | -0.12 |
| 20 | *ITGA2B*.101 | -0.03 |
| 21 | *PDE4C*.059 | 0.12 |
| 22 | *PDE4C*.065 | 0.30 |
| 23 | *PDE4C*.107 | 0.22 |
| 24 | *PDE4C*.119 | 0.64 |
| 25 | *PDE4C*.150 | 0.21 |
| 26 | *PDE4C*.178 | 0.39 |
| 27 | *PDE4C*.259 | 0.29 |

* Target sites correspond to the CpG# in Supplementary Figure S9.

**Table S14. Machine learning model by ElasticNet (swab)**

| | Target sites* | Coefficients |
|---|---|---|
| | (Intercept) | -10.35 |
| 1 | *ASPA.129* | -0.15 |
| 2 | *CCDC102B*.323 | -0.06 |
| 3 | *MEIS1-AS3*.144 | 0.16 |
| 4 | *MEIS1-AS3*.203 | 0.01 |
| 5 | *Elovl2*.052 | 0.63 |
| 6 | *Elovl2*.111 | 0.04 |
| 7 | *Elovl2*.135 | 0.27 |
| 8 | *Elovl2*.149 | 0.26 |
| 9 | *Elovl2*.151 | 0.16 |
| 10 | *Elovl2*.154 | 0.10 |
| 11 | *Elovl2*.202 | 0.0001 |
| 12 | *Elovl2*.233 | 0.03 |
| 13 | *FHL2*.058 | 0.17 |
| 14 | *FHL2*.072 | 0.28 |
| 15 | *ITGA2B*.099 | -0.13 |
| 16 | *ITGA2B*.101 | -0.03 |
| 17 | *PDE4C*.059 | 0.15 |
| 18 | *PDE4C*.065 | 0.27 |
| 19 | *PDE4C*.067 | 0.02 |
| 20 | *PDE4C*.071 | 0.004 |
| 21 | *PDE4C*.093 | 0.02 |
| 22 | *PDE4C*.107 | 0.25 |
| 23 | *PDE4C*.119 | 0.57 |
| 24 | *PDE4C*.150 | 0.19 |
| 25 | *PDE4C*.178 | 0.34 |
| 26 | *PDE4C*.259 | 0.19 |

* Target sites correspond to the CpG# in Supplementary Figure S9.
